# Students’ Experiences of Working With a Socio-Scientific Issues-Based Curriculum Unit Using Role-Playing to Negotiate Antibiotic Resistance

**DOI:** 10.3389/fmicb.2020.577501

**Published:** 2021-01-20

**Authors:** Konstantin J. Sagmeister, Christoph W. Schinagl, Suzanne Kapelari, Pamela Vrabl

**Affiliations:** ^1^Department of Subject-Specific Education, University of Innsbruck, Innsbruck, Austria; ^2^Department of Microbiology, University of Innsbruck, Innsbruck, Austria

**Keywords:** socio-scientific issues, educational strategies, students’ experiences, science and society, antibiotic resistance, science communication

## Abstract

The emergence and widespread of antibiotic-resistant pathogenic microorganisms are of great individual and societal relevance. Due to the complex and multilayered nature of the topic, antibiotic resistance (ABR) is the object of concern for several scientific fields, such as microbiology or medicine, and encompasses a broad range of political, economic, and social aspects. Thus, the issue related to antibiotic-resistant bacterial diseases offers an excellent platform for designing and implementing the teaching and learning of socio-scientific issues (SSI). We created a SSI-based curriculum unit for use in secondary science classrooms by developing a collaborative partnership between education researchers and microbiologists. This classroom environment allows students to explore and negotiate ABR as a societal and scientific phenomenon. For this purpose, we leveraged role-playing within the SSI-based unit as a productive context for engaging students in learning opportunities that provide multiple perspectives on ABR and the complex interplay of its accelerators. This case-based paper describes Austrian school students’ experiences from their participation in a SSI-embedded role-playing classroom environment and subsequent activities that included a mini congress with a poster presentation and a panel discussion. An open-ended questionnaire-based assessment tool was used to examine the situational characteristics of the students’ work. To assess students’ contributions, we applied a qualitative content analysis design and identified cognitive and affective outcomes. The students’ learning experiences demonstrate that they considered the content – the social complexities of antibiotic-resistant bacteria and associated diseases – exciting and very topical. The students perceived that learning about ABR is relevant for their future and involves both individual and societal responsibility for action. Although the curriculum unit and its assignments were described as labor-intensive, it became apparent that the role-playing setting has the potential to inform students about multiple stakeholder positions concerning ABR. Concerning the promotion of science practices, almost all students claimed that they learned to organize, analyze, evaluate, and present relevant information. Moreover, the students affirmed that they learned to argue from the perspective of their assigned roles. However, the students did not clarify whether they learned more through this SSI-based classroom instruction than through conventional science teaching approaches.

## Introduction

Science education and practice aim to support society in acquiring skills that enable citizens to make well-informed decisions and form evidence-based opinions on current societal challenges (Dawson and Venville, 2010; Osborne, 2010). Antimicrobial resistance (AMR) is a pressing and capacious problem in the field of Science|Environment|Health pedagogy (Zeyer and Dillon, 2019). In the coming years, this significant public health issue will progressively gain importance for both public discourse as well as science teaching because of its potential to affect humankind’s personal, social, and global patterns of behavior (Fensham, 2012).

As the primary form of AMR, antibiotic resistance (ABR) occurs when bacteria adapt and increasingly acquire resistance to antibiotic agents to which they were formerly susceptible (Depardieu et al., 2007; Davies and Davies, 2010; Blair et al., 2015). Usually, ABR and AMR, respectively, are a consequence of natural adaptive selection by a genetic mutation (Andersson and Hughes, 2010). It allows bacteria, particularly those frequently found in healthcare settings, to resist the noxious effects of specific antibiotic agents (Alekshun and Levy, 2007). Although numerous previously deadly infectious diseases have turned into non-life-threatening inconveniences in the antibiotic era, this outstanding scientific progress is unfortunately jeopardized (Carlet et al., 2012; Ventola, 2015).

Infections caused by antibiotic-resistant pathogens have become a growing threat to modern public health care that requires action across all economic and societal sectors, from individuals to communities and from hospitals to entire healthcare systems (Carlet et al., 2011; Laxminarayan et al., 2013). As the resistant pathogens might persist in human or animal organisms, ABR endangers the effective management, prevention, and medical procedure of an ever-increasing range of healthcare-associated infectious diseases. Previous research conservatively estimated that AMR is responsible for 700,000 deaths per year globally (O’Neill, 2016). In a recent study, Cassini et al. (2019) reported that more than 33,000 deaths, which were assignable to infections with selected antibiotic-resistant bacteria, occurred in countries of the European Union and the European Economic Area in 2015.

Human activities, such as misuse and overuse of antibiotics, inadequate hygiene precautions, and unfavorable practices in healthcare settings or the food chain, have accelerated the emergence and transmission of drug-resistant pathogens (Carlet et al., 2011). Apart from their every-day usage for clinical purposes in human and veterinary medicine, the misuse of antibiotics as prophylactic protection and growth promoter across industries, such as animal husbandry and aquaculture, has also accelerated the emergence of resistance in many parts of the world (Wassenaar, 2005; Defoirdt et al., 2011; van Boeckel et al., 2019).

Without effective action to reverse current trends, the rise of antibiotic (multi-)resistance can lead to antibacterial agents that are less effective and potentially useless. Resistance to one specific antibiotic agent can lead to resistance to a whole class of antibiotics using a particular functional mechanism (Magiorakos et al., 2012). In addition, the development of new types of antibacterial medicines for clinical use, especially medicines that are effective against multi-resistant strains of bacteria, remains meager (Levy and Marshall, 2004; Brown and Wright, 2016).

These circumstances have triggered the development of coordinated and comprehensive national, European, and global action plans to face this problem. A common goal of these efforts is to improve awareness and understanding of the responsibility for individual and collective actions through effective communication, education, and training to create and promote circumstances for behavioral changes (World Health Organization, 2015). Indeed, guidance for antibiotic usage should be developed, according to Sharland et al. (2018), to meet the United Nations’ Sustainable Development Goals, particularly the aims for good health and well-being (target three). Future generations of scientifically literate antibiotic users need to understand the role that particular stakeholders play in producing, prescribing, and using antibiotics to decrease ABR (World Health Organization, 2015; O’Neill, 2016). Cultural and conceptual knowledge, as well as numerous capacities and skills about ABR, are vital for improving health literacy (Sørensen et al., 2012; Hoffmann et al., 2014) and microbiology literacy (Timmis et al., 2019) in broader society. In conclusion, optimizing antibiotic use to reduce the impact and limiting the spread of resistance, especially multi-resistance, remains manifold and complex.

The rise of antibiotic-resistant bacterial infections affects all members of a community or society and is driven by many interconnected factors. As with other issues of human concern that the media is frequently showcasing, ABR is a significant, open-ended, and multifaceted contemporary societal issue that incorporates many disciplines and knowledge domains. Hence, antibiotic resistance as a global phenomenon offers an excellent entry point for dealing with socio-scientific issues (SSI) instruction in class to contribute to the scientific literacy development of students (Roberts and Bybee, 2014).

Socio-scientific issues classroom instruction represents a science teaching approach that anchors a comprehensive real-world societal issue with conceptual, procedural, or technical links to science as a context for learning (Sadler, 2004). By definition, SSI as curriculum practice entails: (i) participation in dialog, discussion, debate, and argumentation about personally relevant problems through evidence-based reasoning; (ii) the use of evidence from sciences as well as other disciplines to inform decisions; (iii) some degree of moral reasoning and ethical evaluation; and (iv) the development of virtue and character aimed in the long term (Zeidler, 2014).

Research has revealed that teaching about real-world contexts by serving students’ interests and employing personally relevant issues could increase engagement among learners (Sadler, 2009). A substantial body of literature has documented that SSI approaches ought to successfully support students in acquiring desired educational objectives, including interest and motivation in learning science (Albe, 2008; Romine and Sadler, 2016); skills in science practices (Sadler et al., 2007), such as reasoning (Sadler and Zeidler, 2005) and argumentation (Evagorou and Osborne, 2013); and epistemic understandings of science (Eastwood et al., 2012; Khishfe et al., 2017). Despite this progress, “there have been fewer advances in understanding how SSI can be productively incorporated in learning environments,” as Sadler et al. (2017) noted.

**Figure 1:**
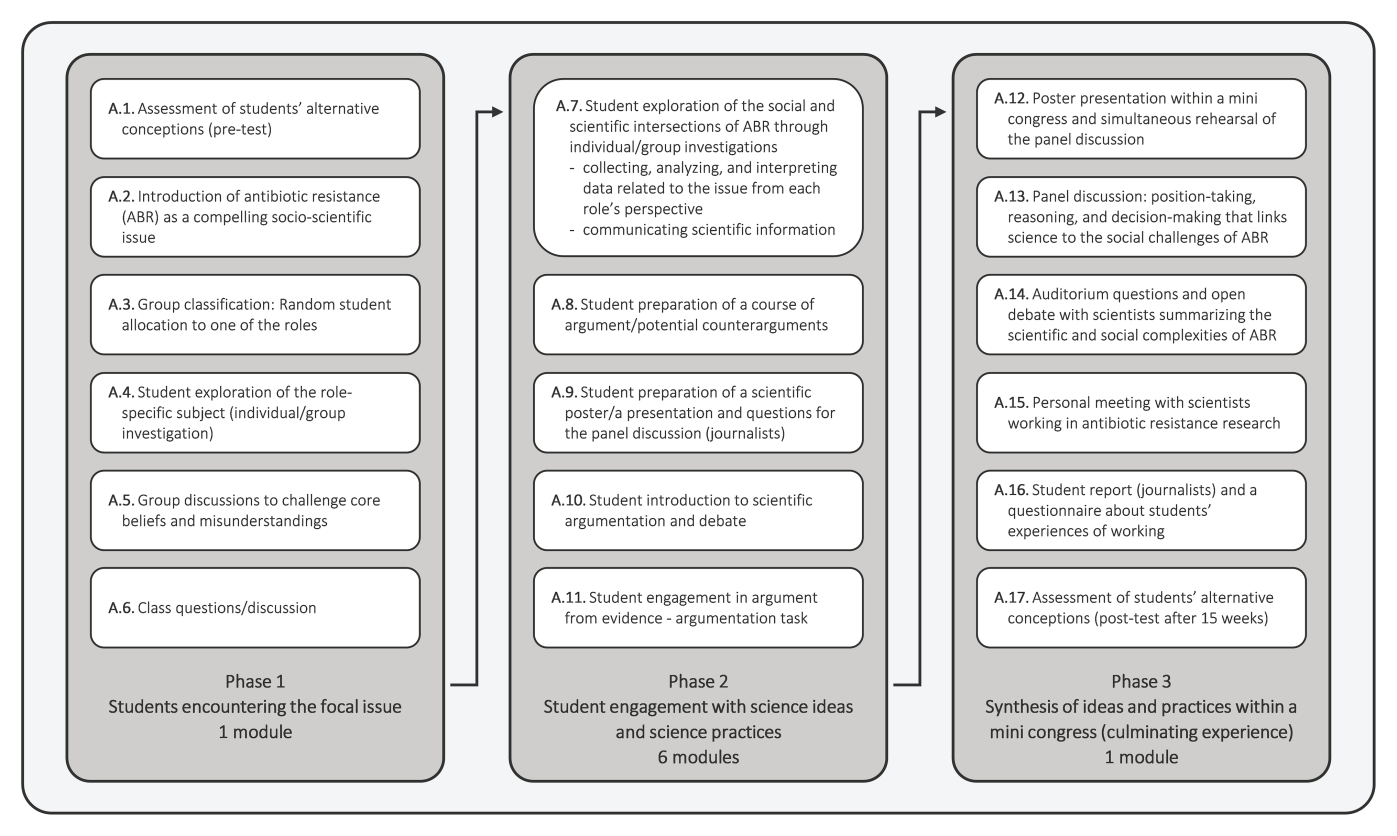
Overview of the classroom procedure of the SSI-embedded role-playing curriculum unit consisting of eight modules. A, activity.

Building on the methodology of “educational design research” (McKenney and Reeves, 2019), we intended to frame learning conditions focusing on engaging students to negotiate the scientific and social connections inherent in a hot spot of public health. To design the implementation of SSI-based instructional activities, we drew from both an SSI framework described by Presley et al. (2013) and a model for SSI teaching and learning proposed by Sadler et al. (2017; see Classroom Procedure section; Figure 1). The SSI teaching and learning instructional model posits three phases (Sadler et al., 2017):

- The first phase involves students exploring the focal issue;
- The second phase corresponds with the main body of teaching and learning experiences, including student engagement with science ideas and higher-order practices, such as argumentation, decision-making, and socio-scientific reasoning (SSR). Following the theoretical construct conceptualized by Sadler et al. (2007), SSR implies typical kinds of reasoning contained in most SSI. Accordingly, SSR consists of four epistemological traits: (i) recognizing the inherent *complexity* of SSI; (ii) examining issues from *varied perspectives*; (iii) appreciating that issues are subject to ongoing *inquiry*; and (iv) possessing *skepticism* in the examination of potentially biased information (Sadler et al., 2007);
- The third and final phase covers a culminating exercise where the students synthesize their learning experiences with the issue under investigation.

As ABR is multidimensional, the issue demands consideration from different perspectives and dimensions. For this purpose, we leveraged role-playing (Howes and Cruz, 2009; see Supplementary Table S2) within our SSI-embedded curriculum unit as a productive context for engaging students in learning opportunities that connect school experiences with a real societal debate. Ødegaard (2003) argues that “the role-play presents a learning opportunity which focuses both on scientific epistemology and scientific personality.” Like an imitation of a societal practice, role-playing exercises have been widely recommended. Scholars and practitioners have revealed numerous advantages of learning through role-play classroom approaches. These advantages include enabling students to potentially gain and improve an understanding of multiple perspectives on issues at both macro and micro levels, enhancing emotional engagement with matters of human concern, and developing and bettering individual and interpersonal skills (Bolton and Heathcote, 1999; Van Ments, 1999; McSharry and Jones, 2000; Simonneaux, 2001; Howes and Cruz, 2009; Belova et al., 2015). The active involvement of students in reinterpreting information and data from a different perspective facilitates a more stable anchoring of the knowledge gained (Duveen and Solomon, 1994).

**Figure 2:**
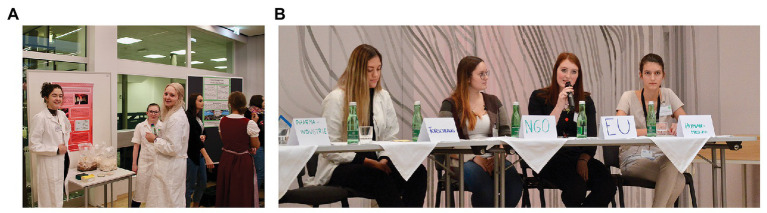
Impressions of the poster presentations at the mini congress **(A)** and the panel discussion **(B)**. **(A)** Three students in lab coats are waiting for visitors to present their poster about novel candidates for antibiotics and displaying on the desk bags and Petri dishes with different fungal cultures as a source of antibiotics. **(B)** Students vividly discussing the crucial factors of antibiotic (multi-)resistance in their respective roles (from left to right): a publicly-funded scientist, an activist of a non-governmental organization, a member of the European Commission, and a physician. Students representing the pharmaceutical industry, the agriculture, the World Health Organization, or the hosting journalist(s) are not depicted in the photograph. For the students’ experiences in the respective roles, see Results section; Table 3.

There is evidence that this learner-centered approach is useful in implementing real-world contexts in science education (Simonneaux, 2001; Agell et al., 2015; Belova et al., 2015), for example, by utilizing role-based panel discussions (Vrabl and Vrabl, 2012). In a role-based panel discussion (RBPD), a variant of a role-play, the students act in place of the assigned position taken in front of an audience as panelists (see Phase 3: Synthesis of Ideas and Practices Within a Mini Congress, Providing a Culminating Experience section; Figure 2B). As student-active tools, role-playing in general and RBPD more particularly raise no claim to a solely clear-cut conclusion of the problem or debate. The emphasis here is not the solution of a problem, but the recognition and understanding of the underlying structural conflict patterns from multiple perspectives (Ødegaard, 2003), such as socio-political dynamics of controversially discussed microbiological-related issues (Vrabl and Vrabl, 2012). However, the application of societally oriented role-playing activities in science classrooms seems to remain limited (McSharry and Jones, 2000; Hofstein et al., 2011).

Educational studies that contextualize ABR lessons related to SSI instruction have often only focused on a single or a few aspects. Teaching and learning about evolution, natural selection, or modeling has been often emphasized (Friedrichsen et al., 2016; Williams et al., 2018; Peel et al., 2019). The present study aims to contribute to this research area by investigating students’ engagement in examining and negotiating scientific and social ramifications inherent in this complex issue. These social dimensions significantly shape the issue and interrelate habitually with how the underlying science is applied or interpreted (Saunders and Rennie, 2013). In this work, we report on students’ accounts of SSI teaching and learning in a role-playing classroom setting, illuminating ABR as a multidimensional and multi-perspective field of health, social, economic, and ecological relationships and discourses. Embedded in SSI-based instruction, the research question guiding this investigation was: How do students experience a role-playing learning environment which addresses the complex interplay of societal and scientific issues related to the phenomenon of antibiotic resistance?

## Materials and Methods

### Ethics Statement

This study was carried out following the recommendations of the Internal Review Board for Ethical Issues of the University of Innsbruck (Austria). Permission for participation and ethical approval of all procedures was obtained and approved by the provincial government’s school authorities, namely the “Bildungsdirektion für Tirol” as well as the “Bildungsdirektion für Vorarlberg,” which are the institutions that approve studies involving school students in the Austrian provinces Tyrol and Vorarlberg. The corresponding ethical approval code is 113.08/0067-allg/2018. A letter of information was provided to parents, guardians, teachers, and the headmasters prior to the classroom activity and the surveys. Signed guardian consent forms were obtained, allowing the students to voluntarily participate in the study with the possibility of withdrawal at any time; no refusals were registered. Parents were prompted to advise the lead researcher if they did not want to disclose sensitive information of their minor child under the age of 18, which happened in three cases (two cases in study group A and one case in study group B). These students did not provide any information concerning age and first language (L1; for the profile of the study groups, see Table 1). The surveys were conducted anonymously to protect student data and privacy. In this article, written informed consent to publish any potentially identifiable images or data was obtained from the students’ guardians and full-age students (aged 18 years or older), respectively. In order to avoid influencing the students’ answers, none of the researchers and project team members were familiar with the participants.

**Table 1:** Number, sex, and age of participants.

Group	Grade level	Number of students	Females		Males		No entry	*M*_age_	*SD*_age_	Age range	No entry
		*n*	*n*	*%*	*n*	*%*					
A	11	26	25	96	1	4	-	17.42	0.58	17–19	2
B^1^	9	26	9	35	17	65	-	14.88	0.78	14–18	1
C^1^	10	19	8	42	11	58	-	15.26	0.45	15–16	-
Total	9–11	71	42	59	29	41	-	15.88	1.31	14–19	3

1 This group participated in the science communication project but was not analyzed in this paper.

### Preliminary Pilot Activities

At a higher education level, we built on experiences with role-playing and the staging of role-based panel discussions for demonstrating socio-political dynamics of controversial topics within microbiology, such as (xeno)estrogens in wastewater (Vrabl and Vrabl, 2012). With the assistance of biology student teachers who were about to graduate, we employed this approach in two pilot projects (held in 2016 and 2017) that engaged senior high school students in the discussion of the controversial topic of ABR.

The pilot activities were performed in two grade 11 biology and environmental education classes from two Upper Secondary Schools (senior high schools) in Tyrol (Austria). Based on the insights and data gained from the pilot classes, which were not used for the analysis of this study, the role-based classroom setting was slightly refined and expanded by a mini-congress with a poster exhibition (see Phase 3: Synthesis of Ideas and Practices Within a Mini Congress, Providing a Culminating Experience section; Figure 1, A.12.; Figure 2). It was deemed essential to rebalance the roles that the students undertook. For example, *university research* was integrated as an independent role because, in the previous distribution of the responsibilities, it was felt that the pharmaceutical industry appeared as the sole innovation driver for new antibiotic substances, which gave the students representing the pharmaceutical manufacturers a certain unassailability. However, universities have made and continue to make decisive contributions to research into novel antibiotic substances, alternative therapies, or resistance mechanisms. Coates et al. (2011) emphasized the role of university research in stemming the tide of ABR and argued that “universities should be encouraged to rebuild their antibiotic discovery sectors and to replace lost skills in this field. Clearly this will take decades, but antibiotic discovery is something that will need to be continued into the foreseeable future.” The unique characteristics distinguishing university research from its competitors, public funding issues, or its underlying structural problems and challenges, all these aspects are subsumed with the redefined role of the *publicly-funded scientist*.

Most of the pre-service teachers and the biology teacher of study group A have been involved in at least one of these pilot interventions. Thus, the project members, i.e., the students’ mentors, were thoroughly acquainted with the procedure and the role-specific subject matters. In the current study, particular emphasis was given to embed the context of SSI teaching and learning into the role-playing classroom environment.

### Classroom Procedure

We describe the curriculum unit using Sadler et al. (2017) model of SSI teaching and learning as a framework. Figure 1 provides an overview of the schedule of the curriculum unit. Supplementary Table S1 presents a lesson plan for the curriculum unit. We designed the role-playing classroom environment to promote the students’ contextual understanding of the scientific and social concepts and processes underlying antibiotic resistance. Each SSI module required two to three consecutive lessons (50 min each) at least once a week, i.e., a total of 16 h of in-class instruction (see Figure 1; Supplementary Table S1). As an additional motivation, students were advised that enrollment in this classroom setting would contribute to their biology grade. The assessments of the students were carried out by their biology teacher.

Groups of three to four students were assigned to examine one ABR role perspective, and each group was accompanied by a mentor, who had thoroughly studied the perspective of a specific role to employ critical analysis and skepticism. Through online student mentoring and personal group meetings, each student group was explicitly able to draw on the support and advice of a supervisor who guided the workflows throughout the curriculum unit. Thereby, the emphasis was placed on (i) advising and supervising the workflow; (ii) instruction for subject-specific questions; and (iii) providing feedback on the quality of the student assignments. The guidance offered to each group aimed to ensure that the students would not be distracted or overwhelmed when working with a complex issue and with an outcome that cannot be predefined.

The class’ biology teacher, a group of scientists, and biology student teachers from the University of Innsbruck offered the participants content knowledge to scaffold student learning and higher-order practices. Supplementary information covered fields of microbiology, immunology, molecular biology, and genetics, tempered with knowledge about the social processes necessary to understand ABR. Subject-specific contents were reinforced and reiterated on multiple occasions, and this was done outside the classroom as well.

This mentoring ensured that the students were generally familiarized with the basic underlying concepts across several levels of biological organization, i.e., cellular, organism, and population levels. More specifically, they were encouraged to develop deeper scientific understandings of the mechanisms of ABR aligning with the social problems and consequences antibiotic-resistance bacteria may cause. Student activities also included a field trip to the Department of Microbiology of the University of Innsbruck before the closing SSI phase for those allocated to the publicly-funded scientists’ group (see Supplementary Table S2).

#### Phase 1: Students Encountering the Focal Issue

The curriculum unit began with presenting a compelling issue as a means of contextualizing the ensuing classroom activities requiring student engagement and commitment to active discovery. As teaching about bacteria, antibiotics, and resistance provides common misunderstandings (Simonneaux, 2000; Gregory, 2009; Byrne, 2011; Brookes-Howell et al., 2012; Bohlin et al., 2018), the alternative conceptions of the students were considered and elicited in this introductory sequence (see Figure 1, A.1.).

During this initial experience with the personally relevant issue, i.e., one module (see Figure 1, A.1.–A.6.), the students were confronted with demonstrations of newspaper headlines, articles, and visual presentations of the current scientific debate to capture their attention (see Figure 1, A.2.). Through formal instruction, the students then were familiarized with and reminded of basic facts and contexts related to bacteria, antibiotics, and resistance. This teaching sequence provided the students with the theoretical background, including a fundamental vocabulary, to enable better comprehension of information obtained through personal investigation and small group negotiation activities.

Eight roles were designed to expose the range of societal intersections involved in the highly complex issue of antibiotic-resistant bacteria (see Supplementary Table S2). Wherever possible, we sought to link the roles to the socio-cultural context of Tyrol/Austria to make the scope of ABR relevant and engaging for the students:

- The publicly-funded scientist (i.e., microbiologists in particular);
- The representative of the pharmaceutical industry (i.e., executives of a drug manufacturing company);
- The livestock farmer;
- The physician (i.e., clinicians and general practitioners);
- The activist of a non-governmental organization (i.e., individuals concerned about the national or international scope of antibiotic policies);
- The representative of a supranational organization (i.e., a panel of the European Commission);
- The international public health official (i.e., a representative of the World Health Organization);
- The journalist.

The students were randomly allocated to one of the roles mentioned above (see Figure 1, A.3.). Subsequently, they conducted first individual and group investigations to explore the role-specific subject matter (see Figure 1, A.4.). As scaffolds, the students received pre-structured information in the form of a role-specific assignment sheet (e.g., for the pharmaceutical industry representatives, see Supplementary Figure S1), assisting them in preparing for their respective roles by providing an evidence base and lines of reasoning. A small collection of selected documents and online sources provided the learners with further information. Supplementary Table S2 illustrates each role’s chain of potential argumentation and selected sources.

Socially shared class activities (see Figure 1, A.5.) were used to challenge the students’ core beliefs and misunderstandings. Class questions and discussion (see Figure 1, A.6.) provided the learners with a place where they can expand on their existing knowledge about the issue, concluding phase one.

#### Phase 2: Student Engagement With Science Ideas, Science Practices, and Socio-Scientific Reasoning Practices

The second phase of the curriculum unit consisted of six modules (see Figure 1, A.7.–A.11.). These modules were intended to promote inquiry to encourage students with science practices that reflect the complex social and scientific intersections. We pursued to encourage students to employ the following practices: (i) collecting, analyzing, and interpreting data; (ii) communicating scientific information by using information and communications technologies; and (iii) engaging in argument from evidence. To allow students to develop better understandings in all areas, we offered the learners occasions to exchange specialist content knowledge and report their findings among their classmates. During the assignments (see Figure 1, A.7.–A.9.), the students were in contact with their mentors, both in person and online.

Each student group was expected to organize and analyze evidence and sources throughout several weeks (see Figure 1, A.7.; Supplementary Figure S1), including evaluating the validity and reliability of evidence, to support the stakeholder position to which their team was assigned. More specifically, the students aimed to properly understand the role they had assumed through investigating, both individually and in groups, media and Internet resources pertaining to the various stakeholders. These sources covered scientific articles, scientific and governmental reports, and presentations of original experimental and epidemiological data prepared for broader audiences.

This exercise (see Figure 1, A.7.) resulted in the formulation of a full course of argument and a chain of potential counterarguments informed by ideas and commitments from each role’s perspectives (see Figure 1, A.8.). Additionally, each learner team created a scientific poster collaboratively illustrating their stakeholder position except for the student group representing the science journalists (see Figure 1, A.9.; Figure 2A). Both assignments (see Figure 1, A.8., A.9.) were aimed to encourage students to develop and deepen their understanding and informed opinions based upon reliable evidence backing each role’s standpoint.

The journalists’ group took a unique position within these two activities (see Figure 1, A.8., A.9.). These students collected the chain of argumentation from each representative group to familiarize themselves with the stakeholders’ standpoints. On this basis, the journalists prepared a presentation for the panel discussion’s opening that highlighted the features of the emergence and dissemination of ABR and the complex interplay of its accelerators. Besides, this learner group developed a list of questions to moderate the RBPD. These questions were intended to point out the contradicting opinions and divergent interests of the panelists.

In phase 2, discussion activities were used to engage students in dialog and support their argumentation and reasoning skills in a module lasting three lessons (see Figure 1, A.10., A.11.). First, the students were introduced to scientific argumentation (see Figure 1, A.10.). The students then had to deal with an oral and written argumentation task, which aimed at encouraging them to apply their reasoning skills and utilize their newly acquired knowledge (see Figure 1, A.11.) reflecting the social and scientific dimensions of antibiotic resistance adapted from Rafolt et al. (2019b).

In the meantime, further school classes were introduced in the subject, explaining the problems related to the spread of antibiotic-resistant bacterial diseases. As congress participants and attendants of the panel discussion (Figure 1, A.12.–A.14.; Figure 2), these students (*n* = 139 in total; study group A: *n* = 48; study group B: *n* = 48; study group C: *n* = 43) constituted the audience for the culminating exercises (see Phase 3: Synthesis of Ideas and Practices Within a Mini Congress, Providing a Culminating Experience section). First, in small groups of three to four, the students were allocated to one of the stakeholder groups (see Supplementary Table S2). As part of a series of structured activities, the students explored data and information related to bacterial diseases and ABR. Next, they prepared questions for the plenary discussion carried out after the penal discussion (Figure 1, A.14.) from the perspective of the relevant stakeholder group. After studying the views of a specific stakeholder group, the students reported their findings and insights to their fellow students.

#### Phase 3: Synthesis of Ideas and Practices Within a Mini Congress, Providing a Culminating Experience

In the final SSI phase (see Figure 1, A.12.–A.17.), students synthesized their learning experiences related to the claims made and corresponding to the questions posed within a mini congress, including a poster presentation (see Figure 1, A.12.; Figure 2A) and a panel discussion (see Figure 1, A.13., Figure 2B). This phase challenged the students to create and justify recommendations for limiting the emergence and spread of antibiotic-resistance with human health risks. The culminating activities were intended to reveal the social dimensions of ABR. The students were able to: (i) demonstrate their awareness of how scientific ideas and practices encountered affect stakeholder group perspectives and (ii) foster decision-making that links science to social challenges. Likewise, students applied their scientific understandings and role-specific knowledge to grapple with some of the societal challenges and problems that emerge from ABR. Figure 2 presents images from the mini congress, including a poster presentation and the closing panel debate experience.

While two to three randomly selected members of each student group presented their scientific posters by communicating scientific information to peers and teachers (see Figure 1, A.12.; Figure 2A), another randomly selected member of each student group was nominated to represent and defend the assigned position in the panel discussion (see Figure 1, A.13.; Figure 2B). These students rehearsed the organizational procedure of the panel discussion before they went into the discussion.

Following the poster presentation, the panel discussion took place (see Figure 1, A.13.; Figure 2B). Requisites (e.g., white coats for the physicians; traditional working garments of Austrian farmers; suits and ties for the politicians; and laboratory coats for the scientists) were provided to facilitate role assignment (see Figure 2). One or two students belonging to the journalists’ group moderated the panel discussion, which started with an introductory presentation about the focal topic (see Figure 1, A.9.). The hosts, i.e., moderators, were then responsible for leading the debate by questioning the panel members, giving the floor to someone, or calling them to order when necessary.

In the panel discussion (see Figure 1, A.13.; Figure 2B), the students outlined antibiotic resistance at an individual, national, or international level from each role’s perspective. More specifically, the discussants negotiated their positions, sometimes illustrated by studies to substantiate their specific area, and debated the responsibility for the current situation of antibiotic-resistant bacteria, explored alternatives, and even sought novel or creative solutions. The discussion ended with a closing statement by each panelist in which they emphasized why their approach to contain the worldwide emergence and spread of ABR may be sensible.

After that, the audience forming students questioned the panel members within an open debate (see Figure 1, A.14.). As ABR constitutes a complex problem that lacks simple, clear-cut solutions, real-life scientists from the field of microbiology summarized the panel discussion and the subsequent plenary questions (see Figure 1, A.14.). These experts highlighted that knowledge of the emergence and spread of antibiotic-resistant bacteria needs to be highly contextualized within a complex social, political, and economic context. After both discussions, all students were given a chance to talk to microbiologists and pharmacologists working in novel antimicrobial substance research (see Figure 1, A.15.). The reason behind this was to provide meaningful, authentic every-day life experiences concerning antimicrobial research.

The students representing the journalists wrote an article summarizing the students’ investigations on the focal issue for a popular science magazine (see Figure 1, A.16.). After 15 weeks, the students repeated the survey to elicit their conceptions on ABR (see Figure 1, A.17.).

### Participants

So far, secondary school (senior high school) students ranging from grades 9 to 11 from three publicly-financed schools located in urban areas in Tyrol (study groups A and B) and Vorarlberg (study group C) participated in the curriculum unit as part of a school-based science communication project. For this work, we analyzed the classroom experiences of the grade 11 students in detail (i.e., group A). Table 1 summarizes the profile of participating study groups. We selected the participant schools due to their particular focus on teaching natural and human sciences. We assumed that the students (age range: 14–19), who have chosen this specialization, have learned comparable subject-specific content at school and are motivated learners. A representative comparison of the samples was not sought. All students participated in the intervention within their regular biology classes (at least 100 min. per week).

In the autumn 2018 semester, the first investigation (study group A) consisted of 26 grade 11 students (aged 17–19, 96% females) from a Secondary School for Economic Professions (College for Higher Vocational Education). Twenty-five female students and one male student with a mean age of 17.42 ± 0.58 years took part in the curriculum unit throughout 8 weeks consecutively. Due to the tradition of the school type in general and the school’s history more specifically, the vast majority of students attending the participating school were girls. German was the first language for all students, but one student spoke Turkish fluently (see Table 1). The school has an influential culture that emphasizes cross-disciplinary teaching with cross-curricular connections and student-orientated lesson design, encouraging students to work and learn in a self-reliant manner. We chose this study group for the implementation of our SSI curriculum unit because: (i) The biology teacher draws on three decades of teaching experience and (ii) took part in previous interventions; (iii) the school directorate immediately embraced the request to collaborate. Furthermore, students from this instructional level (grade 11) were recruited because this is the academic year when ABR-related issues are particularly accentuated (Federal Law Gazette II No. 340, 2015). As the students were planning to take their final exams the following year, we deliberately sought to motivate them to consolidate and enlarge content knowledge and to employ their written and verbal argumentation skills.

### Data Collection

Qualitative data were collected by using a paper-and-pencil task requiring written open-response explanations to assess the situational characteristics of students’ experiences of working with a SSI-based curriculum unit using role-playing to negotiate ABR. Table 2 provides the questionnaire-based assessment instrument with six items. For 2 weeks upon completion of the classroom activities (winter holiday period), the grade 11 students (*n* = 26) were asked to explicitly describe their personal experiences of working with this teaching and learning environment. All students were instructed to answer as completely as possible. Those students who were absent when the collection of student responses took place handed in their open questionnaires later. The biology teacher forwarded the inquiries to the first author to ensure the anonymity of the study participants.

**Table 2:** Questionnaire-based assessment tool to examine students’ experiences of working with the SSI-embedded curriculum unit using role-playing to address antibiotic resistance.

No.	Item^1^
(1)	Describe the *learning experiences* made while being engaged in the SSI-embedded role-based classroom setting addressing antibiotic resistance.
(2)	Describe the *learning progress* made while being engaged in the SSI-embedded role-based classroom setting addressing antibiotic resistance.
(3)	Describe the *learning outcomes* made by participating in the SSI-embedded role-based classroom setting.
(4)	Explain *whether and why* this SSI-embedded role-based setting may or may not be *recommended* to fellow students.
(5)	Give reasons if some *activities* of the SSI embedded role-based curriculum unit might be *changed* if the setting is repeated.
(6)	Describe whether or not the *outcome of the group work* was satisfactory.

1 The questions were administered in German.

### Data Analysis

A qualitative case study design was used to answer the given research question (Stake, 2010). Participants’ contributions were iteratively examined for common features applying an inductive category development (Mayring, 2015). The statements of the students were prepared and examined in accordance with the following four steps:

1. Preparation of raw data: transcription of students’ written essays;
2. Editing the transcripts, i.e., transfer of students’ statements into a grammatically correct form;
3. Arranging and coding students’ testimonies, i.e., a summary of identical or similar statements to thematic groups;
4. Explication, i.e., interpretation of the statements and identification of learning outcomes;

Using the computer-assisted qualitative data analysis software MAXQDA™ (release 20.0.6), the data collected were codified and organized into themes (Rädiker and Kuckartz, 2019). Finally, two other qualitative researchers independently reviewed parts of the transcripts for eliciting the learning experiences of the students. Although it is acknowledged that some students demonstrated particularities in their thinking, we have sought to produce a generalization of the individual student statements. In our attempt to understand the students’ experiences with a learning environment, we found it crucial to go beyond subjective experiences and, therefore, strived to address the collective understanding of student responses. The interpretations are based on the German transcripts; quotations have been subsequently translated. All names have been pseudonymized.

## Results

Inductive text analysis on the written contributions of the grade 11 students revealed cognitive and affective outcomes. Table 3 indicates the frequency of categories of learners’ experiences of working with the SSI-based curriculum unit. According to the students’ self-reported experiences, the majority found the content exciting and related to a current societal challenge. Most students claimed to have learned “a lot,” according to their judgment. As the students stated here, the curriculum unit is useful for learning new facts and generic skills. The unit “offers a variety to the tiring every-day school life,” as Nora emphasized. Elisa also elucidated: “It was interesting to be in such a close contact with scientists and microbiologists and to get to know their job better.”

**Table 3:** Selected categories of students’ experiences in the course of the SSI-based curriculum unit.

Category^1^	Frequency (*n* / 26)
The student recommended the curriculum unit to classroom fellows.	24 / 26
The student indicated to have learned “a lot”.	21 / 26
Exciting and personally relevant content.	20 / 26
Role-playing facilitated the student’s contextual understanding.	19 / 26
The student was satisfied with the group work.	19 / 26
The assignments were labor-intensive.	18 / 26
Peripheral influences affected student engagement.	12 / 26
Student engagement in science practices.	12 / 26
Certain activities required a high degree of student self-organization.	11 / 26
The student referenced individual decision-making regarding antibiotic consumption.	8 / 26
The student reported inconsistencies regarding the division of labor within the student group.	8 / 26
Communicating data and information in front of an audience was a demanding task.	7 / 26
The student struggled in assuming the role.	7 / 26

1 The categories are sorted by the number of students mentioning this experience.

The students noted that the curriculum unit revealed individual and societal as well as national and supranational levels of conflict, such as individual and collective health care decision-making. Addressing the causes and possible solutions to the emergence and spread of antibiotic-resistant bacteria, the students perceived that working with this SSI is relevant for their every-day lives and future. Some students considered to have benefitted from the knowledge gained. Regarding the enhancement of individual health outcomes, students stated that they had become more aware when taking antibiotics. A few students noted that they would refrain from consuming mass-produced meat because it might be contaminated with antibiotic-resistant pathogens.

In large part, students found the experience of working with this classroom approach, which leverages role-playing within SSI teaching and learning, and its assignments labor-intensive. Most of the students underestimated the time required to solve the tasks (see Phase 2: Student Engagement With Science Ideas, Science Practices, and Socio-Scientific Reasoning Practices section; Figure 1, A.7.–A.9.; Supplementary Figure S1). Despite a weeklong working period, some students reported that this interval was insufficient to engage with the subject from various perspectives accurately, deeply, and satisfyingly. That is why students wished for more SSI modules because some struggled to complete all tasks during the lessons offered. Elsa commented: “Such a classroom approach requires much time. The students have to deal with the topic in detail. In any case, sufficient time must be made available in the school lessons to fulfill the required tasks.” Some participating students indicated that peripheral influences affected their engagement in the curriculum unit. They reported that the classroom unit’s implementation coincided with assignments and tests that had to be carried out for other subjects. This circumstance consequently reduced, for some students, the motivation for participating. Therefore, the students demanded to have a say in the selection of the classroom approach’s time of implementation. The willingness of the students to complete assignments out of school was low.

The starting phase (see Phase 1: Students Encountering the Focal Issue section; Figure 1, A.1.–A.6.) was essential for sparking interest and stimulating the search for and evaluation of role-specific data and information. The statements of the participants showed that students found it initially challenging to empathize with the role they took on (see Supplementary Table S2). For example, the students representing the publicly-funded scientists initially struggled in assuming their role because they knew very little about a microbiologist’s profession, as one student elucidated. The visit to the microbiological laboratories at the University of Innsbruck and the interaction with scientists in person facilitated the students in understanding their role. This experience was also relevant for students who had undertaken other roles: the longer they dealt with the topic from their roles’ perspectives, the easier it became to represent the role.

The examination of the subject matters through individual and group investigations (see Figure 1, A.4., A.7.–A.9.), and the taking up of specific roles had a very personal effect. Some students declared that the role-playing supported a secure anchoring of the content knowledge. Others resounded the deep rooting of the knowledge acquired as they expressed that the culminating experiences (see Phase 3: Synthesis of Ideas and Practices Within a Mini Congress, Providing a Culminating Experience section; Figure 2), i.e., the poster presentation (see Figure 1, A.12.; Figure 2A) and the panel discussion (see Figure 1, A.13., A.14.; Figure 2B) in particular, also contributed to the subject matters becoming a longer-term memory. Luca recapitulated: “Role-playing remains in memory.” Students mentioned that they discussed the issue with their families, friends, and acquaintances outside the classroom. These discussions supported the assumption of the role and the elaboration of the assignments.

Observing ABR from different perspectives and dimensions helped the students to develop a proper contextual understanding to face antibiotic resistance, as students emphasized. Role-playing thus facilitated the student’s contextual understanding. Many students claimed that they inquired about several stakeholder positions, highlighting the focal issue’s scientific and social intersections that make the problem challenging. Talking about examining ABR from various perspectives, Florine pointed out that “the role-playing activities provided me with lots of new information on the subject of ABR, both in general and in relation to the individual small groups. The inside views gained from the perspectives of the different roles were extremely informative and gave me a multifaceted idea of the rather questionable use of antibiotics in different areas.” However, a few students argued that they gained role-specific knowledge of ABR predominantly. Prominently, the students referred to the following circumstances on the promotion of ABR:

- The incorrect prescription of antibiotics through physicians;
- The unnecessary ingestion of antibacterial drugs by self-medicated patients;
- The high development costs and the related outsourcing of antibiotic manufacturing to lower its production costs;
- The restriction of antibiotic research conducted in academia due to funding cuts; and
- The unnecessary use of antibiotics in agriculture and husbandry to promote growth or prevent infections and their dissemination into the environment.

They also noted that they acquired an understanding of the social dynamics inherent in the reduction of ABR based on the different interests of various stakeholders. The increase in ABR with limited development of novel antibiotics requires a prudent, controlled, and appropriate use of these substances in all areas of application, as several students summarized.

Students’ learning experiences indicated that the activities engaged them in SSR and higher-order practices, such as argumentation and authentic decision-making based on sound facts. Student work also provided experiences in teamwork, conflict management, and organization. For certain activities, such as student interaction with science ideas and practices (see Phase 2: Student Engagement With Science Ideas, Science Practices, and Socio-Scientific Reasoning Practices section; Figure 1, A.7.–A.9.), it required a high degree of student self-organization and autonomy. They were committed to taking responsibility for themselves and their group members. In some cases, the students reported that it was not easy for them to divide the work evenly among group members. Contacting the group supervisor when needed, provided the students with confidence in completing the assignments. Two students suggested that roles should not be assigned randomly (see Phase 1: Students Encountering the Focal Issue section; Figure 1, A.3.). Instead, students should have the opportunity to allocate the groups by themselves, as Valeria elucidated: “If the groups could be selected freely, there might have been a greater sense of community among classmates. The division of labor might have been fairer. Also, we would certainly be more motivated to work together after school.” However, most students were satisfied with both their own and their group’s achievement. According to several student testimonies, working in groups led to a more comprehensive understanding as opposed to examining SSI through individual investigations because “through group work, we have been able to do a more versatile elaboration of the topic than individually,” as Franziska stated.

The students argued that they learned to organize, analyze, evaluate, and present relevant scientific data and information concerning the promotion of science practices (see Phase 2: Student Engagement With Science Ideas, Science Practices, and Socio-Scientific Reasoning Practices section; Figure 1, A.7.). “For our future professional careers, it was beneficial to practice evaluating the seriousness and credibility of various sources,” as Nora mentioned. However, some students found organizing essential sources, identifying arguments, and judging the credibility and validity of scientific data challenging.

The intensive mentoring enabled the learners to contribute to the group and discussion activities, as several students stated. Others echoed this belief by emphasizing the importance of the group mentor’s assistance, in particular, to establish a scientific poster (see Figure 1, A.9.). The students expressed that elaborating relevant arguments and counterarguments from each role’s perspectives empowered them to argue for the assigned role’s standpoint. The engagement with science and SSR practices helped prepare for the writing of a diploma thesis and the oral “Reifeprüfung” examination, i.e., their final school-leaving oral exams, in the following year.

Student statements showed that the presentation and communication of relevant information in front of an audience within the culminating experience was a demanding task (see Figure 1, A.12., A.13.; Figure 2). Concerning the panel discussion participants’ selection, some students commented on being glad that they did not have to take this assignment and could present their posters instead. One student suggested waiving the random drawing of poster presentation and panel discussion participants. Instead, each student group should choose the roles for themselves in the culminating experience. Three students emphasized that they, for example, managed to get over the fear of communicating data and information to an audience, as they either had to exhibit their poster or participate in the panel discussion. A student panel member, Nadia, proudly described the experience of participating in the panel discussion as follows: “The concluding panel discussion was a new experience. In the beginning, I was very calm and did not worry at all. When we took our places and started discussing, I felt nervous and just wanted to leave. After the discussion started, it was still difficult at first, but it became easier with time. One came fully into the role. At some point, I did not realize that we were sitting in front of many people. Time also passed very quickly. It was a beautiful experience.” According to two student panel discussion members’ experiences, the rehearsal of the procedure also supported a self-confident appearance in the final debate.

Except for two participants, all students recommended this classroom unit to fellow students attending a life sciences specialization. However, the students could not estimate whether they learned more through this SSI-based classroom instruction than through conventional science teaching approaches.

## Discussion

According to the WHO (World Health Organization, 2018), “antibiotic resistance is one of the biggest threats to global health, food security, and development today.” Although resistance occurs naturally, the misuse and overuse of antibiotics in human and animal health care is accelerating the problem. Antibiotics are often given without professional oversight, are taken by people with viral infections, are given as growth promoters in animals, or are used to prevent diseases in healthy animals (Ventola, 2015). Thus, one of the five strategic objectives in the “Global Action Plan on Antimicrobial Resistance” is to improve awareness and understanding of this phenomenon to promote prudent antibiotic usage (World Health Organization, 2015).

In the last years, information and educational campaigns have been launched in several countries to increase antibiotic awareness (Cross et al., 2017). Waaseth et al. (2019) argued that “public knowledge is considered a prerequisite for appropriate use of antibiotics and limited spread of antibiotic resistance.” In a recent study, Burstein et al. (2019) systematically identified public-directed interventions to promote antibiotic awareness in the United States. They found that multifaceted programs can change patient perspectives regarding antibiotic use. However, “most public messaging interventions focused on educating parents of young children through office-based posters and handouts,” as they concluded (Burstein et al., 2019).

In the case-based study described by this paper, microbiologists and educational researchers came together to address the focal problem in the context of SSI teaching and learning (Presley et al., 2013; Zeidler, 2014; Sadler et al., 2017). In terms of the pedagogical practice, we designed an 8-week extended SSI-based classroom setting to engage students in examining and negotiating the scientific and social relationships of ABR (see Classroom Procedure section; Figure 1). We leveraged role-playing within the curriculum unit to illustrate controversial perspectives and dimensions of multiple stakeholders that habitually shape this deeply ramified issue with connections to science and society. To understand whether the curriculum unit successfully meets the goal of conveying ABR’s multilayered interrelationships between social, political, economic, and scientific perspectives and dimensions, we drew on the methodology of “educational design research” (McKenney and Reeves, 2019). Retrieved from an open-response questionnaire (see Data Collection section; Table 2), grade 11 students’ experiences of working with our SSI-based classroom unit using role-playing were qualitatively examined.

The present classroom intervention addressed juveniles and young adults in particular who could become parents, physicians, scientists, farmers, politicians, or could walk any other path of life. These individuals will likely be asked in the future to decide how and when to use antibiotics. By then, they may consider lessons learned and may make informed decisions. Many students argued that they learned “a lot” about antibiotics, the patterns for the emergence of antibiotic resistance, and different stakeholder perspectives on ABR. However, there is evidence that after 16 h of dealing with the focal issue, few students still have difficulties in explaining who is getting resistant (humans or bacteria) or how it happens that antibiotics become less effective in treating bacterial infections (Brookes-Howell et al., 2012). Despite a high level of knowledge of antibiotics and ABR among citizens, “there seems to be a knowledge gap when it comes to understanding the rationale behind the resistance problem,” as Waaseth et al. (2019) similarly highlighted.

The evolution of ABR is multifactorial and deeply rooted in societal practice and individual ideas and beliefs. Thus, learning opportunities that shed light on the complexity of the phenomenon are needed in order to raise public awareness and proper conceptual understanding. Educational interventions designed by scientists, governmental organizations, or Centers for Disease Control often rely on traditional approaches. They predominantly address the scientific rationale that underlies the phenomenon while focusing on a particular goal, such as decreasing unnecessary antibiotic use or prescription (Burstein et al., 2019).

Hardly ever do educational interventions address links between science and society or allow learners to situate their actions in a broader societal context. Real-life intersections between science and society have been identified as SSI within the science education community already (Zeidler, 2014). Previous research has documented that SSI teaching and learning can have a positive impact on the science content learning of students (Klosterman and Sadler, 2010), on their understanding of the nature of science (Khishfe et al., 2017), and on their argumentation (Romine and Sadler, 2016), and the development of critical thinking skills (Sadler et al., 2007; Rafolt et al., 2019a). Overall, Hancock et al. (2019) emphasized that “SSI-based instruction has emerged as an effective way for students to contextualize their science learning within a complex social and political context.” However, the implementation of SSI-based teaching in the every-day classroom remains limited because SSI instruction may be unfamiliar for many teachers (Ekborg et al., 2013). Besides, teachers frequently believe that their content knowledge is limited, and they experience a paucity of well-designed SSI-oriented curricular materials as well as limited support while trying to enact SSI teaching (Presley et al., 2013).

Socio-scientific issues curriculum units are challenging not only when it comes to designing a learning environment but also equally when the curriculum unit is put into practice. To overcome these barriers, microbiologists, pre-service biology teachers, and education researchers designed a SSI-embedded curriculum unit using role-playing (see Classroom Procedure section; Figure 1), including a plenary discussion within a mini congress setting (see Phase 3: Synthesis of Ideas and Practices Within a Mini Congress, Providing a Culminating Experience section; Figure 1, A.12.–A.14.; Figure 2). The role-playing classroom environment encouraged students to scrutinize the individual positions advocated and their dynamics with one another. It created identification and empathic understanding (Ødegaard, 2003). In providing meaning to content, role-playing allows the students to slip into a previously unknown position and deal intensively with their thoughts, attitudes, and interests, which may be of a private, public, political, or commercial nature concerning ABR. The designed roles (see Supplementary Table S2) represent diverse and multiple perspectives related to the emergence and spread of antibiotic-resistant bacteria, supporting students to learn more meaningfully about the relevant content. However, not everyone approves the task to put himself/herself into a role. Students of 17–19 years of age (see Table 1) have little or no experience with their assigned or other stakeholder professions, their daily tasks, or challenges. This unfamiliarity may make it difficult to either support or reject a specific routine in prudent antibiotic use. Hence, these students need increased support and detailed information on their role characteristics.

This observation led us to another prerequisite of successful SSI learning environments. A broadly based support system is helpful, not only for students but for teachers alike. Microbiologists, pre-service teachers, and education researchers supported participating teachers and students while being engaged in the classroom setting. The intensive mentoring enabled students to contribute substantially to the group and discussion activities. The group mentor’s assistance was urgently needed to design a scientific poster and elaborate relevant arguments and counterarguments for each role’s perspectives. Microbiologists introduced content knowledge, while education researchers offered a theoretically substantiated selection of teaching and learning tools. Teachers shared their practical expertise in scaffolding the student learning processes in-class. In this context, the question arises to what extent SSI learning approaches are feasible to be implemented on a broader scale or whether it should be a sustainable endeavor to support intensive collaboration between researchers and schools.

Students’ self-reports about how they experienced working with SSI instruction are mainly positive (Ottander and Ekborg, 2012). However, Hancock et al. (2019) argued that the local and national contexts, school routines, and cultural traditions might have a considerable impact on student performance. All students reported that they had to take exams in several subjects while preparing for the poster presentation and plenary discussion. Accordingly, the time devoted to specific tasks (see Classroom Procedure section; Figure 1, A.7.–A.9.) was limited. Nevertheless, most students wished to have time to do more SSI modules and deepen their knowledge about ABR. The interactive learning environment helped students thrive and evoked their interest and motivation to learn more about ABR. However, the main obstacle that was difficult to overcome was linked to the time constraints of the students. Therefore, we highly recommend being aware of “Peripheral Influences” (Presley et al., 2013) that may also influence student learning in SSI-based units.

Overall, this study contributes to the enhancement of SSI teaching and learning in a real-world context with microbiology. In the study described for this paper, microbiologists and educational researchers presented a learning environment that has the potential to improve students’ awareness and understanding of ABR. Microbiology offers a useful perspective and contributes to real-life contexts for SSI teaching and learning. Topics relevant to health and environmental education are tied together, and a significant challenge of the 21st century is negotiated (Fensham, 2012; Zeyer and Dillon, 2019). Concerning the case-based data reported, this work supports the assumption that our SSI-embedded role-playing classroom setting is well suited to support students in acquiring scientific knowledge about antibiotics and ABR and the complex interplay of social dimensions. However, Sjøberg and Schreiner (2012) have shown that there are gender differences in students’ interests in learning about science topics, their experience with and views on school science, and their views and attitudes to science in society. In this study, mainly girls participated. The attitudes of males to this intervention might differ from what is reported. There are limitations caused by using an open question paper-and-pencil format. Students may interpret given questions differently and have varying competencies to express themselves in writing and drawing. Unfortunately, it was not possible to address long-term knowledge retention because participants were not available for interviews at any time later. However, this could be rewarding data to use in the subsequent use of this curriculum unit. Accordingly, additional research is required to assess whether the curriculum unit can help students develop higher-order thinking skills, such as argumentation, decision-making, or position-taking. Future research might also explore the extent to which students’ SSR competencies improve while being engaged in the curriculum unit (Romine et al., 2020).

## Data Availability Statement

The raw data supporting the conclusions of this article will be made available by the authors, without undue reservation.

## Author Contributions

PV conceptualized and designed the classroom unit together with CS. PV raised the funding. KS performed the study and gathered the data. KS wrote the first draft of the manuscript, which then was critically revised and rewritten by CS, SK, and PV. All authors contributed to the drafting of the figures, data analysis and interpretation, and read and approved the final version of the manuscript.

### Conflict of Interest

The authors declare that the research was conducted in the absence of any commercial or financial relationships that could be construed as a potential conflict of interest.

## Abbreviations

ABR: Antibiotic resistance
AMR: Antimicrobial resistance
L1: First language
SSI: Socio-scientific issues
SSR: Socio-scientific reasoning
RBPD: Role-based panel discussion
WHO: World Health Organization
