# Supplementary material for: Students’ Experiences of Working With a Socio-Scientific Issues-Based Curriculum Unit Using Role-Playing to Negotiate Antibiotic Resistance
Source: Front Microbiol. 2021 Jan 20;11:577501. doi: 10.3389/fmicb.2020.577501 (PMC7855855; doi:10.3389/fmicb.2020.577501)
Supplement: Supplementary file 1 [file Data_Sheet_1.pdf]

Supplementary Table S1. Lesson plan for the SSI-based curriculum unit addressing antibiotic resistance. Each SSI module required two to three consecutive lessons (50 min.) at least once a week.

Phase	Module	Lessons	Instructional focus	Lesson plan for the classroom session
Students encountering the focal issue	1	2	Introducing antibiotic resistance (ABR) as a compelling, significant, and relevant socio-scientific issue (SSI).<br>Eliciting students' alternative conceptions.<br>Explaining bacterial evolution, natural selection, and mechanisms of ABR.<br>Student exploration of one ABR role perspective.	Welcoming and introduction of the group supervisors.<br>Administer pre-test to assess students' alternative conceptions.<br>Demonstration of newspaper headlines, articles, and visual presentations (as part of a MS PowerPoint-Presentation).<br>Formal instruction (lecture): bacteria, natural selection, antibiotics, resistance.<br>Random student allocation to one of the eight roles.<br>Distribution of the role-specific assignment sheets.<br>Presentation and discussion of the work assignments (1-4).<br>Individual and group investigation to explore the subject matters (partially in the school's computer room).<br>Group discussion to challenge students' core beliefs and misunderstandings.<br>Class questions/discussion.
Student engagement with science ideas, science practices, and socio-scientific reasoning practices	2-6	2	Encouraging students with science practices, science ideas, and socio-scientific reasoning practices.<br>Promoting students' contextual understanding of the scientific and social concepts and processes underlying ABR.<br>Communicating scientific information.	Collection, analysis (evaluation of the validity and reliability of evidence), and data interpretation related to the student role.<br>Communication of scientific information through socially shared class activities to exchange role-specific content knowledge among classmates.<br>Students formulate a full course of argument and a chain of potential counterarguments.<br>Students create a scientific poster, which illustrates the role's perspectives on ABR.<br>Students develop a list of questions to moderate the panel discussion's opening (journalists' group).<br>Students prepare a presentation for the panel discussion (journalists' group).

	7	3	Promotion of argumentation skills	Introduction to scientific argumentation and debate.<br>Students engage in argument from evidence: oral and written argumentation task, adapted from Rafolt et al. (2019b).
Synthesis of ideas and practices within a mini congress (culminating experience)	8	3	Synthesis of ideas and practices within a culminating experience.<br>Fostering student position-taking, reasoning, and decision-making that links science to social challenges.<br>Student demonstration of their awareness.	Students present their posters within a mini congress to fellow students and concurrent rehearsal of the panel discussion (with the student panel members and student hosts).<br>Student panel discussion.<br>Plenary questions/discussion moderated by scientists.<br>Student interaction with scientists to provide authentic every-day life experiences.<br>Students elaborate a press release for a popular science magazine about the activities and experiences made (journalists' group).
			Eliciting students' alternative conceptions.	Administer post-test to assess students' alternative conceptions.

Note: Between module seven and module eight, two additional activities were held, partially with other school classes: (a) further school class students, which constituted the audience for the culminating exercise, were introduced to the topic and explored the problems related to the spread of ABR as part of a series of structured activities (one lesson per class; in total three classes). Besides, those students prepared questions for the plenary discussion carried out after the student panel discussion; (b) a field trip to the Department of Microbiology of the University of Innsbruck (Austria) was organized for the students allocated to the publicly-funded scientists' group to provide them in-depth insights into antimicrobial research and microbiologists' profession.

Supplementary Table S2. Description of the roles' chain of potential argumentation along with selected documents and online sources.

Student role	Description of the student role along with key considerations and standpoints	Selected sources related to the role's perspective on ABR
Publicly-funded scientist (i.e., microbiologists in particular)	Almost all approved antibiotics are derived from antibiotically active substances formed by bacteria or fungi. Therefore, new antibiotics could emerge from the search for undiscovered microbial natural substances. Academic research institutions may be involved in all stages of drug development. Due to funding cuts, antibiotic research conducted in academia has been scaled back. Researchers are gathering knowledge about the course of bacterial infections, processes in bacterial cells, and mechanisms of how resistances develop and spread. This research may provide indications of new targets for bacterial infections and ways to prevent or overcome resistance. Experts assume that only a small part of the existing bacteria and fungi have been cultivated in laboratories. Besides, many of these in such a manner that they do not even show which antibiotics they form. However, innovative cultivation and examination methods allow the study of microorganisms for novel antibiotic substances in biological resources (e.g., bacterial or fungal cultures). The government-funded scientists are convinced that the emergence or advancement of innovative antibiotics or alternative practical therapeutic approaches such as antimicrobial photodynamic therapy, phage therapy, or metagenomics need to be researched to fight antibiotic resistance more extensively. For a possible way out of the rising threats from antimicrobial resistance, public funds are urgently needed to expand the scope of research and development activities on this pressing issue significantly.	Golkar, Z., Bagasra, O., and Pace, D. G. (2014). Bacteriophage therapy: a potential solution for the antibiotic resistance crisis. *Journal of Infection in Developing Countries*, 8(2), 129–136. doi: 10.3855/jidc.3573<br>Ventola, C. L. (2016). The Antibiotic Resistance Crisis: Part 1: Causes and Threats. *Pharmacy and Therapeutics*, 40(4), 277–283.<br>Piddoc, L. J. V. (2012). The crisis of no new antibiotics–what is the way forward? *Lancet Infectious Diseases*, 12(3), 249–253. doi: 10.1016/S1473-3099(11)70316-4<br>Ledford, H. (2015). Promising antibiotic discovered in microbial 'dark matter', *Nature*, doi:10.1038/nature.2015.16675<br>Newspaper articles with role-specific content matters of Austrian/German media houses
Representative of the pharmaceutical industry (i.e., executives of a drug manufacturing company)	The pharmaceutical industry is increasingly withdrawing from antibiotics research internationally. The development of new antibiotics by pharmaceutical companies, a strategy that had been effective at fighting resistant bacterial strains in the past, had mostly stalled because of economic and regulatory obstacles. Besides, business combinations between pharmaceutical corporations have also substantially reduced the number and diversity of antibiotic research teams. The announcement that the production at the Kundl (Tyrol, Austria) site, the	Barlett, J. G., Gilbert, D. N., and Spellberg, B. (2013). Seven ways to preserve the miracle of antibiotics, *Clinical Infectious Diseases*, 56(10), 1445–1450. doi: 10.1093/cid/cit070<br>Sukkar, E. (2013). Why are there so few antibiotics in the research and development pipeline? *The Pharmaceutical Journal*, 291, 520. doi: 10.1211/PJ.2013.11130209

	only penicillin production facility in Europe, would be terminated, made far-reaching headlines in Austria and beyond. Antibiotic development seems to be no longer considered an economically wise investment for the pharmaceutical industry. Many pharmaceutical companies are outsourcing manufacture to Asia. This outsourcing is due to, for example, high development costs, resulting from a limited indication, such as the classification of new active substances as an antibiotic of last resort and the short prescription period of antibiotics in contrast to other drugs. These circumstances may lead to bottlenecks in the supply of particular antibiotics and a drop-in in quality standards. Pharmaceutical companies are therefore dependent on partners who shoulder the economic risks and burdens with them.	Hubert, J., Nuzillard, J.-M., and Renault, J.-H. (2015). Dereplication strategies in natural product research: How many tools and methodologies behind the same concept? *Phytochemistry Reviews*, 16(1),55–95. doi: 10.1007/s11101-015-9448-7<br>Newspaper articles with role-specific content matters of Austrian/German media houses
Livestock farmer	Bacterial infectious diseases may occur in animals. The pathogens in humans and animals are alike, so that most antibiotic groups are suitable for both organisms. By using antibiotics in veterinary medicine, modern agriculture (i.e., intensive livestock farming) promotes the emergence and spread of antibiotic-resistant microorganisms. According to the Austrian Animal Welfare Act, a livestock farmer is obliged, if necessary, to consult a veterinarian and arrange for treatment of sick animals. Treatment of bacterial infections with antibiotics is also carried out in order to avoid economic damage (i.e., the total loss due to the death of the animal; temporary or permanent loss of performance; the danger of the disease spreading in the herd and beyond to other herds; and as a result endangering the health of consumers). However, antibiotics are also used to prevent the spread of expected bacterial infections. Examples of such antibiotic use are the simultaneous treatment of diseased and not (yet) diseased but probably already infected animals. In 2017, over 40 tons of antibiotics for veterinary use were sold in Austria. Furthermore, many antibiotics are only metabolized partially in the human and animal body. Most are excreted with feces and urine and may continue to enter the environment in an active form. Here, antibiotics are found mainly in surface waters and soil. Also, high concentrations can be measured in liquid manure, widely applied as fertilizer to agricultural land. An additional pathway to the environment results from aquacultures. Mass livestock farming thereby contributes to the spread of antibiotic-resistant germs. Residues of antibiotics and resistant bacteria may be present in food (e.g., meat	Van Boeckel, T. P., Brower, C., Gilbert, M., Grenfell, B. T., Levin, S. A., Robinson, T. P., Teillant, A., and Laxminarayan, R. (2015). Global trends in antimicrobial use in food animals. *Proceedings of the National Academy of Sciences of the United States of America*, 112(18), 5649–5654. doi: 10.1073/pnas.1503141112<br>AGES – Austrian Agency for Health and Food Safety Ltd.: Data regarding antibiotic resistant germs in food; antibiotic resistance in livestock; use of antibiotics in livestock farming; sales volumes in veterinary medicine; and measures for the containment of use of antibiotic in livestock farming<br>AGES – Austrian Agency for Health and Food Safety Ltd.: Monitoring of antibiotic resistance in zoonotic pathogens and commensal bacteria in food-producing animal populations and their foods<br>AURES – Resistance reports for Austria (e.g., 2015)<br>Newspaper articles with role-specific content matters of Austrian/German media houses

	and vegetables/cereals) and may be ingested by humans when consuming meat. Recently, molecular detection methods have demonstrated that resistant bacteria in farm animals reach consumers through meat products. These bacteria can cause infections in humans that may lead to adverse health consequences.	
Physician (i.e., clinicians and general practitioners)	Physicians are frequently deciding on the use of antibiotics. Several studies indicate that clinicians and general practitioners prescribe the vast majority of therapeutic antimicrobials, in particular antibiotics. There is cumulative evidence that physicians sometimes unnecessarily prescribe antibiotics. Mostly, this is not due to a lack of relevant knowledge. Instead, factors such as tiredness and patients' or parents' insistence on treatment with antibiotics become decisive. Incorrectly prescribed antibiotics thus contribute to the promotion of resistant bacteria and expose patients to potential complications of antibiotic therapy. Antimicrobial stewardship and vaccination education, both in human and veterinary medicine, can be an effective preventive measure against several bacterial infectious diseases.	Hughes, J. M. (2011). Preserving the lifesaving power of antimicrobial agents. *Jama*, 305(10), 1027-1028. doi: 10.1001/jama.2011.279<br>Hoffmann, K., Ristl, R., Heschl, L., Stelzer, D., and Maier, M. (2014). Antibiotics and their effects: what do patients know and what is their source of information? *European Journal of Public Health*, 24(3), 502–507. doi: 10.1093/eurpub/ckt112<br>NAP-AMR – National Action Plan on Antimicrobial Resistance (Austria)<br>AGES – Austrian Agency for Health and Food Safety Ltd.: Data, sales volumes, and reports on antibiotic resistance<br>AGES – Austrian Agency for Health and Food Safety Ltd.: Antibiotic use and surveillance – Monitoring in hospitals<br>AURES – Resistance reports for Austria (e.g., 2015)<br>Metz-Gereck, S., Maieron, A., Strauss, R., Wieninger, P., Apfalter, P., and Mittermayer, H. (2009). Ten years of antibiotic consumption in ambulatory care: Trends in prescribing practice and antibiotic resistance in Austria. *BMC Infectious Diseases*, 9 (61), doi: 10.1186/1471-2334-9-61<br>Kruse, E.-B-, and Dettenkofer, M. (2010). Epidemiology of and preventive measures for multiresistant pathogens, *Ophthalmologe*, 107(4), 313–7. doi: 10.1007/s00347-009-2074-2<br>Newspaper articles with role-specific content matters of Austrian/German media houses
Activist of a non-governmental organization (i.e., individuals concerned about the national or	The antibiotics used in livestock and aquaculture are ingested by humans when they consume meat products. Recently, the Austrian National Reference Laboratory for Antibiotic Resistance (Austrian Agency for Health and Food Safety Ltd., AGES), showed that every third piece of conventional, i.e., non-organic produced, pork meat purchased in the food retailing is contaminated with antibiotic-resistant	NAP-AMR – National Action Plan on Antimicrobial Resistance (Austria)<br>Reports of non-governmental organizations (e.g., Environment Agency Austria, Greenpeace, Global2000) on antibiotic resistance

international scope of antibiotic policies)	pathogens. These resistant bacteria can cause diseases in humans that may lead to adverse health consequences. A breeding ground for the development and spread of antibiotic-resistant germs is mainly factory farming. Animals are crammed together in small spaces, stand on an unnatural ground, and inflict wounds on each other. These conditions often make the animals sick. Correspondingly, the use of antibiotics in pork factory farming is high. About 74 % of all antibiotics used in Austrian livestock forming are administered to pigs. According to the activities of a non-governmental organization, i.e., individuals concerned about the national or international scope of antibiotic policies, the use of antibiotics in human and veterinary medicine must be limited to what is medically necessary. Otherwise, resistance will develop and spread, rendering antibiotic treatments for bacterial diseases ineffective. The activists demand the reduction of antibiotic usage, for example, in livestock farming through (i) better husbandry conditions; (ii) abolition of metaphylaxis, i.e., control treatment of a group of animals after the diagnosis of infection and clinical disease in part of the group; (iii) abolition of the "right to dispense" for veterinarians, the ban of quantity discounts for veterinary medical products, and the introduction of minimum prices; (iv) the prohibition or restriction of the use of second-line antibiotics (reserve antibiotics) in animal husbandry. Therefore, they demand the launch of an ambitious and binding plan from the European Commission, respectively, the responsible Austrian Ministry, to reduce antibiotics in livestock farming. Besides, Austrian farmers must be supported in the transition to better animal husbandry.	Newspaper articles with role-specific content matters of Austrian/German media houses
Representative of a supranational organization (i.e., a panel of the European Commission)	Combating antibiotic resistance requires a holistic approach involving many different sectors, including human and veterinary medicine, research, livestock and agriculture, environment, trade, and communication). The European Commission's 2011 Action Plan to counter the growing threat of ABR includes 12 actions to be implemented jointly with European Union Member States. This action plan identifies seven areas where the action is most needed. In June 2017, the European Commission reaffirmed the importance of incentive systems for developing new antibiotics with its updated 'European Union One Health Action Plan against Antimicrobial Resistance', i.e., encompassing human health, animal health, and the environment. The	Eurosurveillance - Europe's journal on infectious disease surveillance, epidemiology, prevention and control: Data and reports on antibiotic resistance<br>EFSA – European Food and Safety Authority and ECDC – European Centre for Disease Prevention and Control: European Union summary report on antimicrobial resistance in zoonotic and indicator bacteria from humans, animals, and foods<br>McNulty, C. A. M., and Johnson, A. P. (2008). The European Antibiotic Awareness Day. *Journal of Antimicrobial Chemotherapy*, 62, 853–854. doi:10.1093/jac/dkn410

	plan consists of three main pillars: (i) making the European Union a best practice region; (ii) boosting research, development, and innovation; and (iii) shaping the global agenda. The representatives of the European Commission aim to substantially reinforce existing good practices and support the European Union Member State Austria in preventing the further spread of resistance and preserve the ability of antibiotics to combat antibiotic-resistant bacterial infections (i.e., to put the Austrian national action plan and strategy against ABR in place that is aligned with the objectives of the global action plan).	Horizon 2020 Research and Innovation programme: contributing to public health and tackling antimicrobial resistance<br>European Centre for Disease Prevention and Control (ECDC) (2017). EU Guidelines for the prudent use of antimicrobials in human health. Stockholm: ECDC.<br>European Centre for Disease Prevention and Control (ECDC): Data and reports on antibiotic resistance<br>European Medicines Agency (EMA): Data on sales of antibiotics<br>Guidelines for the prudent use of antimicrobials in veterinary medicine (2015/C 299/04).<br>Newspaper articles with role-specific content matters of Austrian/German media houses
International public health official (i.e., a representative of the World Health Organization)	For many years, the World Health Organization (WHO) and other international institutions have recommended that antibiotic research be jointly advanced. Therefore, the WHO has been leading multiple initiatives to address antibiotic resistance, such as the World Antibiotic Awareness Week, The Global Antimicrobial Resistance Surveillance System (GLASS), or the Global Antibiotic Research and Development Partnership (GARDP). Besides, the Organization for Economic Co-operation and Development (OECD) also recommended a threefold approach for increasing research and development on resistances and antibiotics: (1) promotion of basic research in academic institutions as well as small and medium-sized enterprises, which then flows into a 'G20 global collaboration platform' as a knowledge hub; (2) to support promising development projects against WHO-prioritized pathogens; and (3) seek suitable funding opportunities that make the development of antibiotics more economically attractive and decouple investment in research and development from the need for refinancing from product sales. The WHO is collaborating closely with the Food and Agriculture Organization of the United Nations (FAO) and the World Organization for Animal Health (OIE) in a so-called 'One-Health' approach. The target of 'One-Health' is to promote best practices to prevent and reduce resistance, including optimal antibiotic use in both humans and animals. As public health officers, students assist in helping countries develop their national actions plan and strengthen their health and surveillance	O'Neill, J. (2016). Tackling Drug-Resistance Infections Globally: Final Report and Recommendations.<br>World Health Organization (WHO) (2015). Global action plan on antimicrobial resistance. Geneva: WHO.<br>World Health Organization (WHO) (2014). Antimicrobial resistance: global report on surveillance. Geneva: WHO.<br>World Health Organization (WHO): Fact sheet antimicrobial resistance<br>World Health Organization (WHO): Fact sheet antibiotic resistance<br>Global Antimicrobial Resistance Surveillance and Use System (GLASS): Data and reports on antibiotic resistance<br>Organization for Economic Co-operation and Development (OECD) (2016). Antimicrobial Resistance – Policy insights.<br>Organization for Economic Co-operation and Development (OECD) (2016). Health at a Glance: Europe 2016: State of Health in the EU.<br>World Bank (2016). Drug-Resistant Infections – A threat to Our Economic Future.<br>Newspaper articles with role-specific content matters of Austrian/German media houses

	systems. Coordinated action is required to minimize the increase of ABR.	
Journalist (i.e., a panel discussion moderator).	Science and research on various topics may determine the public discourse as journalists report on scientific content and institutions in various media. The journalists examine (i) the nature of expertise: (ii) the diffusion of knowledge; and (iii) the communication of science and technology among professionals and to the public related to ABR. The journalists' task is to critically accompany the development of ABR-related knowledge from an external perspective, analogous to the journalist's control function in other fields such as politics or economics. Just like journalists in general, they see themselves as neutral and not guided by interests.	Kirchner, S., Springer, B., Su, Y.-S., Fuchs, R., Fuchs, K., Reisenzein, H., Persen, U., and Allerberger, F. (2017). Use of antibiotics in Austria. *Die Bodenkultur: Journal of Land Management, Food and Environment*, 68(1), 17–27. doi: 10.1515/boku-2017-0002.<br>Hoffmann, K., Wagner, G., Apfalter, P., and Maier, M. (2011). Antibiotic resistance in primary care in Austria – a systematic review of scientific and grey literature. *BMC Infectious Diseases*, 11, 330. doi: 10.1186/1471-2334-11-330<br>Ventola, C. L. (2016). The Antibiotic Resistance Crisis: Part 1: Causes and Threats. *Pharmacy and Therapeutics*, 40(4), 277–283.<br>NAP-AMR – National Action Plan on Antimicrobial Resistance (Austria)<br>AURES – Resistance reports for Austria (e.g., 2015)<br>Newspaper articles with role-specific content matters of Austrian/German media houses

Supplementary Figure S1. Example of an assignment sheet from the SSI-based curriculum unit addressing antibiotic resistance. The sheets were administered in German.

Antibiotic Resistance
Pharmaceutical Industry

Within the curriculum unit, you will take on the role of a **representative of the pharmaceutical industry**, i.e., an executive of a drug manufacturing company. Your goal is to represent their views on antibiotic resistance (ABR). To explore multiple resources related to your role's perspective on ABR, you will work in small groups of about four people. To discuss and argue accurately, you will carry out the assignments listed below with your group members. A group mentor is guiding you throughout the unit.

Work assignment (1-4)

1. Read the information material provided. Discuss within the group **critical statements** found in the texts. Write them down and look for relevant sources of information (refer to 2).
2. Use the document provided and search on the Internet to work out **sound arguments** that underline your position on the topic. Note these sources of information (e.g., institution, author, URL) accurately. Keep in mind to consider the quality and credibility of information, especially those that involve scientific data (refer to your group mentor for some helpful tips concerning the credibility and reliability of information/sources). Collect possible counter-arguments of other interest groups and discuss how you can react to those.
3. Consider how you would like to introduce yourself to the panel discussion (tip: what is your expertise?). Formulate a short **introductory statement** illustrating your position on the issue.
4. Create a **scientific poster** using the style sheet provided to represent the pharmaceutical industry's position on antibiotic resistance within a mini congress. The poster should inform and sensitize the mini congress' audience about the current circumstances of antibiotic research in the pharmaceutical industry.

Chain of potential argumentation

- The search for novel antibiotics and their development to marketability swallows up enormous amounts of money (tip: find out the average development costs of an antibiotic).
- Rapidly developing resistance, largely driven by misguided and unreflective handling of these life-saving drugs in society, may make even newly developed antibiotics obsolete.
- Novel antibiotics are very likely to be used only rarely at the beginning (tip: reserve antibiotic), i.e., they find correspondingly short sales, leading to a low turnover for the pharmaceutical company.
- In contrast to drugs for cancer treatment of cholesterol-lowering drugs, antibiotics are often only administered once and for a short time (tip: research costs).
- The majority of the pharmaceutical industry has withdrawn from antibiotic research in the last decades. Why? Which interventions could encourage the pharmaceutical industry to do more research in this area to bring products to the market?
